# Complementary fMRI and EEG evidence for more efficient neural processing of rhythmic vs. unpredictably timed sounds

**DOI:** 10.3389/fpsyg.2015.01663

**Published:** 2015-10-27

**Authors:** Nienke van Atteveldt, Gabriella Musacchia, Elana Zion-Golumbic, Pejman Sehatpour, Daniel C. Javitt, Charles Schroeder

**Affiliations:** ^1^Department of Educational Neuroscience, VU University, Amsterdam, Netherlands; ^2^Institute Learn!, VU University, Amsterdam, Netherlands; ^3^Department of Cognitive Neuroscience, Faculty of Psychology and Neuroscience, Maastricht University, Maastricht, Netherlands; ^4^Department of Audiology and Speech and Language Pathology, University of the Pacific, San Francisco, CA, USA; ^5^Department of Otolaryngology, Head and Neck Surgery, Stanford University Medical School, Stanford, CA, USA; ^6^The Gonda Center for Multidisciplinary Brain Research, Bar Ilan University, Ramat Gan, Israel; ^7^Cognitive Neuroscience and Schizophrenia Program, Nathan S. Kline Institute, Orangeburg, NY, USA; ^8^Department of Psychiatry, Columbia University, New York, NY, USA; ^9^New York State Psychiatric Institute, Columbia University, New York, NY, USA

**Keywords:** EEG, fMRI, temporal context, sound processing, rhythm, auditory cortex

## Abstract

The brain’s fascinating ability to adapt its internal neural dynamics to the temporal structure of the sensory environment is becoming increasingly clear. It is thought to be metabolically beneficial to align ongoing oscillatory activity to the relevant inputs in a predictable stream, so that they will enter at optimal processing phases of the spontaneously occurring rhythmic excitability fluctuations. However, some contexts have a more predictable temporal structure than others. Here, we tested the hypothesis that the processing of rhythmic sounds is more efficient than the processing of irregularly timed sounds. To do this, we simultaneously measured functional magnetic resonance imaging (fMRI) and electro-encephalograms (EEG) while participants detected oddball target sounds in alternating blocks of rhythmic (e.g., with equal inter-stimulus intervals) or random (e.g., with randomly varied inter-stimulus intervals) tone sequences. Behaviorally, participants detected target sounds faster and more accurately when embedded in rhythmic streams. The fMRI response in the auditory cortex was stronger during random compared to random tone sequence processing. Simultaneously recorded N1 responses showed larger peak amplitudes and longer latencies for tones in the random (vs. the rhythmic) streams. These results reveal complementary evidence for more efficient neural and perceptual processing during temporally predictable sensory contexts.

## Introduction

The temporal dynamics of environmental sounds can be predictable, such as approaching footsteps, or unpredictable and novel, such as a tire screech or a notification of a text message. It is increasingly clear that the brain is well-equipped to use predictable, or “rhythmic,” temporal structures within sensory information. It has been shown that ongoing oscillatory brain activity in the macaque auditory and visual cortices can be entrained by rhythmic, task-relevant event streams, such that the cortical representation of the events in that stream is enhanced (Kayser et al., 2008; Lakatos et al., 2008, 2009). Oscillatory entrainment is thought to be metabolically efficient (Buzsáki and Draguhn, 2004) and is sometimes referred to as a *rhythmic mode* of processing (Schroeder and Lakatos, 2009). As many environmental stimuli such as biological motion are inherently rhythmic, characterized by slow rates of 1–5 Hz, this “rhythmic” mode is thought to be beneficial for processing such natural stimuli by enhancing and resetting low-frequency (delta and theta-band) spontaneous oscillations (Schroeder and Lakatos, 2009).

There is also evidence from human research that a phase-resetting mechanism operating in a rhythmic context can increase processing efficiency; resulting in enhanced detection (Fiebelkorn et al., 2011; Ten Oever et al., 2014) and neuronal responses (Thorne et al., 2011; Romei et al., 2012; see discussion in Lakatos et al., 2013b). For example, Thorne et al. (2011) showed that phase-reset elicited by a visual stimulus increased the probability of auditory target detection when a subsequent auditory input arrived within the anticipated time window of optimal processing. Furthermore, Romei et al. (2012) showed that visual cortex excitability co-cycled with sound-induced occipital alpha-phase concentration.

A rhythmic processing mode as described above may not be beneficial in every situation, as it produces relatively long periods of low neuronal excitability during which novel sounds will be harder to detect. While many natural sounds have a rhythmic component to them, the temporal structure may often vary in rhythmicity or rate over time, such as in spoken language, a property which would necessitate adapting to changes in the temporal structure (Schroeder et al., 2008; Zion Golumbic et al., 2012; Henry et al., 2014). In addition, task demands and dynamics determine the relevance and usage of temporal structure, which adds to the necessity to flexibly orchestrate the optimal processing strategy (Van Atteveldt et al., 2014). One theory of how optimal processing may be managed flexibly includes switching between “vigilance” and “rhythmic” attentional modes (Schroeder and Lakatos, 2009). For example, sound detection in an unpredictable context may engage a mode of “vigilant” listening where neuronal excitability in auditory cortex is continuously high, rather than fluctuating between high- and low-excitability states. Maintenance of high excitability is thought to be accomplished by suppression of low frequency and related enhancement of gamma oscillations, both of which are metabolically demanding (Niessing et al., 2005; Kann, 2011; Galow et al., 2014). The role of gamma frequencies during vigilance states is supported by studies showing that shorter response latencies in vigilance tasks such as change detection correlate with higher amplitudes of gamma activity, which has been found in monkeys (Womelsdorf et al., 2006) as well as humans (Koch et al., 2009).

In sum, it is likely that rhythmic-mode processing is efficient in terms of metabolic demands, but is not optimal for perception in all contexts. In the current study, we explored the proposition that the brain flexibly switches between vigilant and rhythmic processing modes depending on the temporal context, and that both behaviorally and at the neural level, detecting target sounds in a rhythmic context is more efficient. We used a combination of electro-encephalography (EEG), functional magnetic resonance imaging (fMRI) and behavioral measures to test our predictions.

Electro-encephalography has proven to be an excellent method to investigate neural efficiency in different contexts. Several lines of research suggest that the event-related potential (ERP) N1 component is particularly appropriate to help index neuronal efficiency in vigilant and rhythmic sound conditions. The N1 is the first, large negative-going peak of an auditory-evoked response. It is generated in primary auditory cortex (Campbell et al., 2007), functions in onset detection, stimulus-specific (Näätänen et al., 1988) as well as complex feature analysis (Banai et al., 2005; Johnson et al., 2007) and is related to both brainstem processing (Musacchia et al., 2008) and conscious perception of sound (for review, see Näätänen et al., 2011). Related to temporal context and flexibility the N1 peak amplitude is smaller when sound onset is predictable, as in a rhythmic context (Parasuraman and Beatty, 1980; Parasuraman and Mouloua, 1987; for review, see Sanders, 2013), and gets larger with task conditions requiring voluntary attention and increased detection difficulty (Hillyard et al., 1973; Näätänen and Michie, 1979; for review, see Näätänen et al., 2011). These data also support the now classic inverse relationship of “neural efficiency” such that more intelligent subjects exhibit lower energy consumption and cortical activation (Haier et al., 1992), which is assumed to be reflected by smaller EEG and ERP amplitudes. According to previous studies and theories, we predict that sounds presented in a rhythmic context will be more efficiently processed, resulting in easier target detection and smaller N1 peak amplitudes, compared to sounds presented at random time intervals.

We measured fMRI simultaneously with EEG to be able to localize brain regions exhibiting differences in processing sounds in the rhythmic and vigilance contexts with great accuracy. Furthermore, the hemodynamic response measured with fMRI (the blood oxygenated level dependent or BOLD response) reflects energy use by neurons (Huettel et al., 2009) and therefore provides a measure of processing efficiency that is complementary to the N1 amplitude measure. As the fMRI signal strength (most probably) reflects energy use of localized neuronal populations, it provides complementary support for the presumed different efficiency of variations in cortical activity between rhythmic and vigilance conditions. Thus, we predict that both fMRI signal strength and N1 amplitude should be smaller under rhythmic conditions.

## Materials and Methods

### Task and Experimental Design

Eleven healthy adults participated in the study (mean age 30.6; age range 23–44; four males). All participants had normal or corrected-to normal vision, and normal hearing as confirmed by hearing thresholds obtained using an audiometer (Earscan 3 Manual audiometer, Micro Audiometrics Corp, Murphy, NC, USA). Participants performed an auditory oddball detection task during the EEG–fMRI recordings, which took place at the Center for Advanced Brain Imaging (CABI) at the Nathan S. Kline Institute for Psychiatric Research (NKI) in Orangeburg, NY, USA. All procedures followed were approved by the CABI Protocol Review Committee and the NKI Institutional Review Board. Informed consent was obtained from all participants prior to the measurements.

**FIGURE 1:**
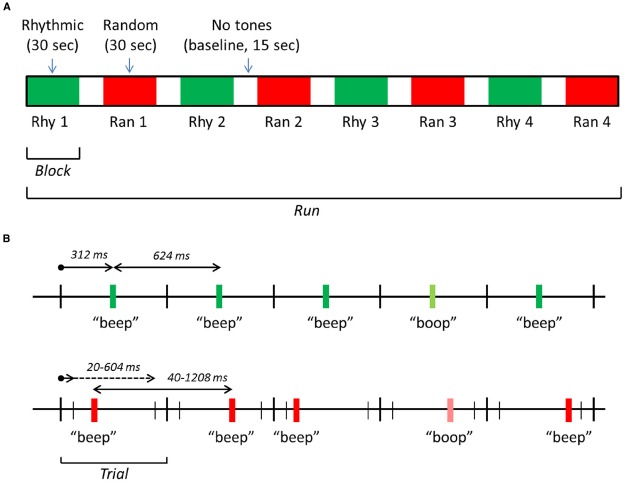
**Schematic representation of the stimulation design. (A)** An exemplar run, consisting of eight blocks: four with rhythmic tones, four with random tones. **(B)** The timing of tones in the rhythmic 1.6 Hz (upper time scale) and random 1.6 Hz (lower scale) blocks. In rhythmic blocks, tones were presented at a regular SOA of 624 ms. In random blocks, tones were presented at a random onset time (interval 20–604 ms after trial onset), producing an irregular SOA between 40 and 1208 ms and a mean SOA of 624 ms. “Beeps” are standard tones (440 Hz) and “boops” target tones (5–10% lower in frequency). Note that the overall structure was the same in the 2.2 Hz driving rate condition, but the exact times were different: the SOA was 454 ms during rhythmic blocks, and between 20 and 434 ms during random blocks.

The software package Presentation®(Neurobehavioral systems, Inc., Berkeley, CA, USA) was used for stimulus presentation, response logging and synchronizing the stimuli with the fMRI scanning pulses. Standard stimuli consisted of 440 Hz pure tones, 30 ms in duration and a 5 ms rise/fall time (Audacity, Inc.). Subjects had to detect infrequent targets (17% of the stimuli) with a lower frequency. The number of standards between two subsequent target tones was 2–8 (randomly selected during the experiment). Target frequency was selected according to a just-noticeable-difference (JND) approximation performed in the scanner, the difference between standard and target frequency was in the range of 5–10%. Tones were presented in blocks of 30 s, with baseline periods of 15 s in between. In half of the blocks, tones were rhythmic (with equal inter-stimulus intervals), in the other half randomly spaced (with randomly varied inter-stimulus intervals). Each run consisted of eight blocks in total, four random blocks and four rhythmic blocks, in alternating order (Figure 1A). The beginning of a block was cued with a brief sound (a high-pitched beep), 2 s prior to the first tone of a block, to avoid a state of vigilance during the baseline periods.

The average driving rate (the rate at which tones were presented) in random blocks was equal to the rhythmic rate: 1.6 Hz in two runs, 2.2 Hz in the two other runs. These driving rates were selected on the basis of a pilot experiment using the same oddball detection task (*n* = 6) and for theoretical reasons: the range of 1–2 Hz (delta-range) contains the frequencies of many relevant environmental rhythmic events (Schroeder and Lakatos, 2009). Starting condition (Ran, Rhy) and driving rate (1.6, 2.2 Hz) of runs were counterbalanced.

For the rhythmic blocks, the 1.6 Hz driving rate was implemented by presenting the tones with a fixed stimulus onset asynchrony (SOA) of 624 ms. Tones were presented in trials of 624 duration, one tone in each trial (see Figure 1B). In rhythmic blocks, tones were presented at exactly 312 ms after the start of the trial. In the random 1.6 Hz blocks, the onset of a tone was randomly selected from an interval of 20 ms to 604 ms after the start of the trial. This produced an average SOA of 624 ms. In the 2.2 Hz runs, trial duration was 454 ms. For the rhythmic blocks, the tones were presented at exactly 227 ms after the start of the trial. In the random 2.2 Hz blocks, the onset of the tones was randomly selected from an interval of 20–434 ms after the start of a trial, producing an average SOA of 454 ms.

### Behavioral Data Analysis

We analyzed the reaction times (RTs) to correctly detected targets (hits), and the ratio of correctly detected targets vs. total amount of targets (hit rates, HRs). Reaction time and HR were used as dependent variable in variables in two-way analyses of variance (ANOVA’s) with condition (Ran vs. Rhy) and driving rate (1.6 vs. 2.2 Hz) as independent variable.

### EEG Recordings and Analysis

Electroencephalographic data were recorded using an MR compatible system developed to record EEG during continuous fMRI scan acquisitions (BrainVision MR series, Brain Products, Munich, Germany). A 64-channel MR-compatible ring electrode cap with 10–20 International System electrode placement cap (including an electrocardiogram, ECG, electrode on the back) was applied to the subject prior to entrance into the magnet. Electrode to scalp contact was adjusted until impedance was <10 kOhm at each electrode. The vertex electrode was used as the online reference and data were sampled at 1000 Hz with a bandpass filter of 0.1–250 Hz applied online. Once in the MRI scanner, impedance was rechecked and electrode contact adjustments were made if necessary. Offline, scanner and heartbeat artifacts were removed with the native BrainVision artifact removal procedures in which a template scanner and heartbeat artifact are constructed and the pattern of that template is removed from the data via application of an inverse matrix (e.g., singular value decomposition). Following this decomposition, data were exported to a MATLAB compatible format. Independent component analysis was performed using the infomax algorithm to identify eye movement artifact (fieldtrip toolbox). Components that exhibited an eye movement pattern (e.g., strong activity in frontal eye channels) were removed from the data and portions of remaining movement-related artifacts were rejected by visual inspection.

For ERP averaging, the data were low-pass filtered at 55 Hz and epoched with a pre- and post-stimulus time window of –150 to 600 ms, respectively. Inspection of the data at this point revealed an intermittent 6 Hz electrical noise artifact that was evident in six out of 10 of the subjects which was a result of loose cap cabling within the magnet. Rejection of this intermittent noise by eye in the continuous data helped to greatly reduce the prevalence of this in the accepted EEG. Individual ERP averages were then created with an artifact rejection criterion of ±150 μV. Channels with >±2.5 SD from the mean amplitude in the post-stimulus time (0–600) were examined visually, and if found to be noisy, were excluded from further analysis. For one participant, we only acquired fMRI data, so the EEG analyses were performed on 10 participants.

In order to determine putative differences in the auditory-evoked potential across conditions, a bootstrap analysis with false discovery rate (FDR) correction for multiple comparisons (α < 0.001) was applied to fronto-central electrodes (Fpz, Fp1, Fp2, Fz, F1, F2, F3, F4, Fc1, Fc2, Fc5, Fc6, Cz, C3, C4, Pz). Following the establishment of ERP differences, global field power (GFP) was then calculated from all of the individuals good electrodes, using the EEGLAB process based on the Lehmann and Skrandies (1984) formula. N1 peaks in the individual GFP averages were picked automatically at maximum values between 115 and 145 ms. Visual inspection of the automatic detection was performed to confirm that the automatic marker placement fell on a peak. Once latency and amplitude values were obtained, student’s paired *t*-tests were conducted to determine the relative latency and amplitude in the rhythmic and random conditions.

It is important to note that at the 2.2 Hz rate there was no clear N1, rather a steady-state response with merged N1-P2 peaks. As we have clear hypotheses concerning the N1, we decided to focus the ERP analysis on the 1.6 Hz-rate. The reason to include two different rates was to enable generalization of the results, and the other measures (fMRI, behavioral) show that the general effects of ran vs. rhy are in the same direction for the two driving rates. To make sure that the 1.6 Hz rate is not affected by similar problems of the merged N1-P2 peaks, we have run the analyses with a sub-sample of the random trials with longer SOA’s (>300 ms).

### fMRI Acquisition and Analysis

Scanning was performed on a Siemens Tim Trio scanner with 12-channel head coil. A gradient-echo EPI (echo planar imaging) sequence was used with the following parameters: TR 2000 ms, TE 30 ms, FA 80°, FOV 240 mm, matrix 96 × 96 (in-plane resolution 2.5 mm × 2.5 mm), slice thickness 3.6 mm, gap 0.9 mm, 25 slices. Intra-session anatomical scans were acquired in each participant using an MPRAGE sequence with 192 sagittal slices and voxel size of 1 mm × 1 mm × 1 mm. Images were analyzed using BrainVoyager QX (Brain Innovation, Maastricht, Netherlands; Goebel et al., 2006). Preprocessing included slice timing correction, 3D motion correction, high-pass filtering using GLM-Fourier (two cycles) and spatial smoothing (FWHM = 8 mm). Functional slices were co-registered to the anatomical volume using position parameters from the scanner and intensity-driven fine-tuning, and transformed into Talairach space. For data presentation, an averaged anatomical volume was created from the 11 individual anatomical volumes.

The functional time-series were analyzed using a whole-brain, random-effects general linear model (GLM) with predictors Cue, Rhy, and Ran. Predictor time-courses were adjusted for the hemodynamic response delay by convolution with a double-gamma hemodynamic response function. To get an overview of brain regions activated during the sound detection task, we first created maps using the second-level contrast of (Rhy or Ran) vs. baseline. To test which brain areas responded differently during the random and rhythmic blocks, we performed the second-level contrast of Ran vs. Rhy, masked with the positive map of the first contrast (i.e., restricted to voxels in which Rhy or Ran > baseline). To correct for multiple comparisons, we corrected the map of the first contrast (Rhy or Ran vs. baseline) using the FDR (Genovese et al., 2002) at Q(FDR) < 0.05. For the second contrast (Ran vs. Rhy), we used cluster-extent thresholding: maps thresholded at an initial voxel-level *p*-value were submitted to a whole-data correction criterion based on the estimate of the map’s spatial smoothness and on iterative Monte Carlo simulations for estimating cluster-level false-positive rates (Forman et al., 1995; Goebel et al., 2006). After 1,000 iterations, the minimum cluster-size corresponding to a corrected false positive probability of <0.05 was applied to the statistical maps. From the clusters in resulting corrected maps, we extracted individual beta-estimates for the Ran and Rhy conditions.

The second analysis (Ran vs. Rhy) was complemented with an ROI-based GLM in which we modeled the eight blocks of each run as separate predictors. This GLM consisted of nine predictors (Cue, Rhy1, Rhy2, Rhy3. Rhy4, Ran1, Ran2, Ran3, Ran4) and was used to extract estimates of fMRI signal change (beta estimates) for each block, from the regions revealed by the whole-brain Ran vs. Rhy contrast.

## Results

### Behavioral Results

**FIGURE 2:**
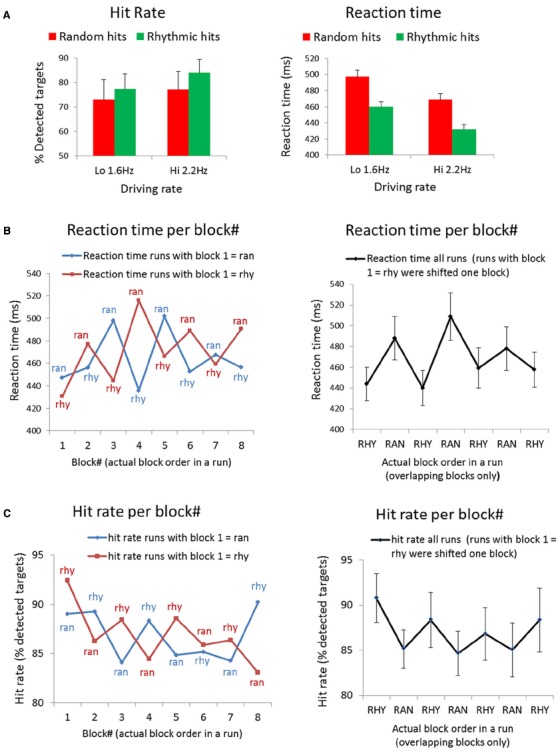
**Behavioral results. (A)** Mean hit rates and reaction times for all targets in the experiment, separated for condition (i.e., targets occurring in rhythmic vs. random blocks) and driving rate (i.e., targets occurring in runs with 1.6 vs. 2.2 Hz sound streams). **(B)** Mean reaction times to targets in each block of a run (block 1–8, see Figure 1A). Left panel: reaction times per block, separate for runs starting with rhythmic (red line) and random (blue line). Right panel: reaction time averaged across all runs. Note that half of the runs (the runs starting with rhythmic) were shifted to match conditions, and only the seven blocks for which all four runs in each subjects overlapped are shown. **(C)** Mean hit rates (% detected targets) in each block of a run (block 1–8; see Figure 1A). Left panel: hit rates per block, separate for runs starting with rhythmic (red line) and random (blue line). Right panel: hit rates averaged across all runs. Note that half of the runs (the runs starting with rhythmic) were shifted to match conditions, and only the seven blocks for which all four runs in each subjects overlapped are shown.

Analyses of the behavioral responses reveal a RT and HR advantage for targets in rhythmic streams (Figure 2A). Using HR as dependent variable, we found a main effect of Condition [Rhy > Ran: *F*(1,10) = 5.95, *p* < 0.05]. The main effect of Driving rate was non-significant [Hi vs. Lo; *F*(1,10) = 3.38, *p* < 0.1], and neither was the interaction of Condition and Driving rate [*F*(1,10) = 0.99, *p* < 0.5]. Two participants showed very low HRs, especially for the random condition (24 and 34%, whereas the mean HR was 75% for random). This may indicate that the JND procedure to determine the target did not work well for these participants and that the target was too difficult for them. Because the mean RTs for these subjects would be based on very few hits (only one or two hits in some of the runs), we excluded these subjects from the RT analysis. Using RT as dependent variable, we found main effect of Condition [Ran > Rhy: *F*(1,8) = 7.35, *p* < 0.05] and a main effect of Driving rate [Lo > Hi: *F*(1,8) = 5.9, *p* < 0.05], the interaction between Condition and Driving rate was non-significant [*F*(1,8) = 0.001, *p* < 1.0].

To examine how RT’s and HR’s evolved over the course of a run, we also looked at average RTs and HRs per block. As displayed in Figure 1A, there were eight blocks in each run: four random blocks and four rhythmic blocks, and the runs were counterbalanced with regard to which condition started. We plotted the RT per condition for blocks 1–8 in Figure 2B (left graph) separately for the runs that started with a random and runs that started with a rhythmic block. Figure 2C (left graph) shows the same for HRs per block. To be able to average RTs and HRs per block for all the runs together, we shifted half of the runs (the runs with rhythmic as first block) to match the conditions. The averaged RT across all runs are shown in Figure 2B (right graph), the averaged HR’s across all runs in Figure 2C (right graph). Note that we only show the blocks that overlapped for all four runs in each subject (seven blocks in total).

### fMRI Results

**FIGURE 3:**
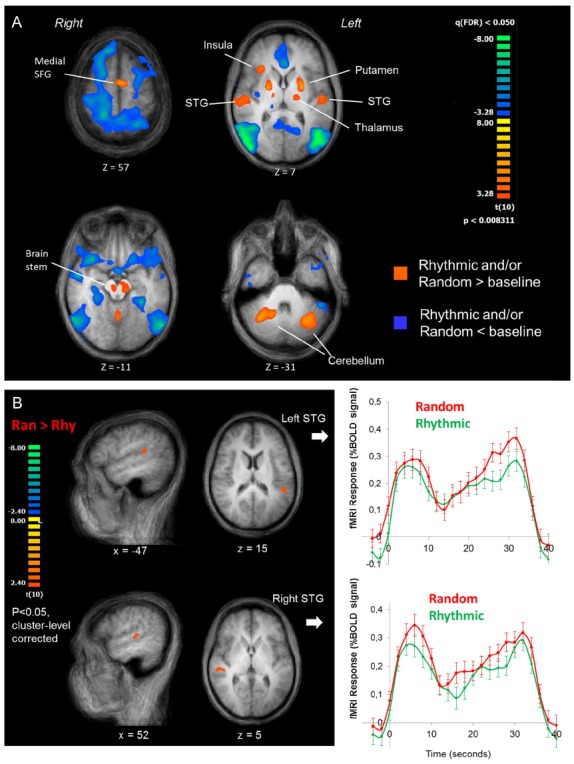
**fMRI results. (A)** Overall pattern of fMRI activation during the sound detection task. The orange map shows voxels where the fMRI response during either task condition was greater than during baseline, the blue map shows voxels where it was lower than baseline. The maps are shown in four transversal slices of the average anatomical image of all participants, going from the top (top left slice) to the base (lower right slice) of the brain. **(B)** Rhythmic vs. Random comparison. Left panel: cluster-level corrected maps of the whole-brain Random vs. Rhythmic contrast, shown in the averaged anatomical image of all participants. Two clusters on the superior temporal gyrus were revealed, one in the left (upper images) and one in the right hemisphere (lower images). Right panel: averaged BOLD response time courses for the random (red lines) and rhythmic (green lines) conditions (averaged at the block onset).

The fMRI results indicate that during the sound detection task, a distributed network is recruited including superior temporal gyrus (STG) bilaterally, insula, superior frontal gyrus (SFG, medial), the thalamus, the brain stem and the cerebellum (Figure 3A, orange map). In addition, several brain regions seem suppressed during task performance relative to the baseline periods (Figure 3A, blue map).

Within the positively activated network, the Ran vs. Rhy contrast revealed a higher BOLD response in the STG bilaterally during random blocks compared to rhythmic blocks (Figure 3B). The averaged fMRI time course in both STG regions over the course of a block shows an interesting dynamic. The BOLD signal initially increases, followed by a drop in signal at ∼10 s, after which it increases again more gradually. Nonetheless, throughout the entire block the BOLD signal is stronger during the random blocks vs. the rhythmic blocks.

### fMRI-Behavioral Correspondence

**FIGURE 4:**
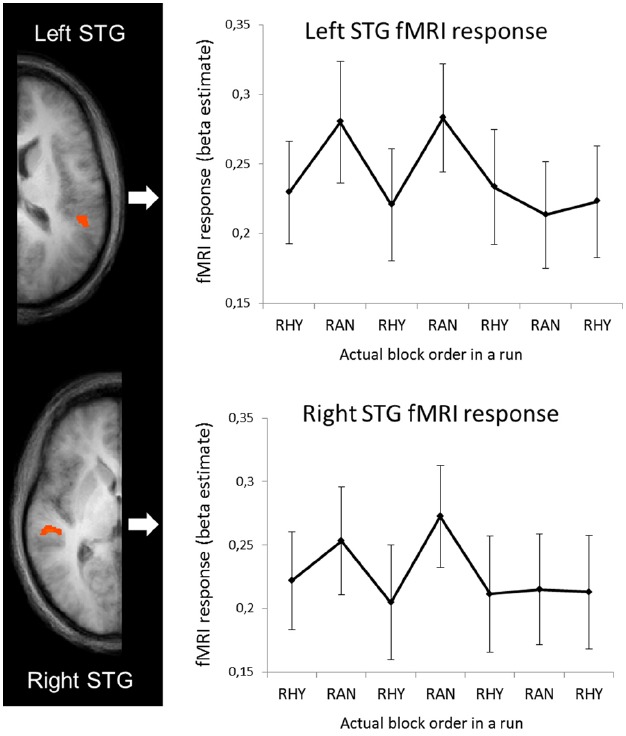
**fMRI response (beta estimates from left and right STG) for separate blocks.** In the graphs, beta estimates for random and rhythmic blocks were averaged across all runs. Note that half of the runs (the runs starting with rhythmic) were shifted to match conditions, and only the seven blocks for which all four runs in each subjects overlapped are shown.

To examine how the fMRI activity changed over the course of the runs, we ran an ROI-based GLM on the averaged time-series of the left and right STG clusters (shown in Figure 3B) with separate predictors for the eight blocks in each run (see Figure 1A, for schematic explanation of blocks vs. runs). The beta estimates for each block are shown in Figure 4. Note that as in Figures 2B,C (right graphs), we shifted half of the runs to match the conditions, and only show the seven blocks that overlapped across all four runs in each subject. To test how well the RTs and fMRI response strength corresponded, we performed bivariate correlation analyses between the fMRI beta estimates in the left and right STG against RT. We took all blocks as separate cases and as RT’s were non-normally distributed [*D*(264) = 0.145, *p* < 0.001], we performed non-parametric correlation tests (Spearman’s rho). For RT and right STG beta’s the correlation was significant (*r*_s_ = 0.26, *p* < 0.001), for RT and left STG beta’s it was not (*r*_s_ = 0.03, *p* < 0.56).

### EEG Results

**FIGURE 5:**
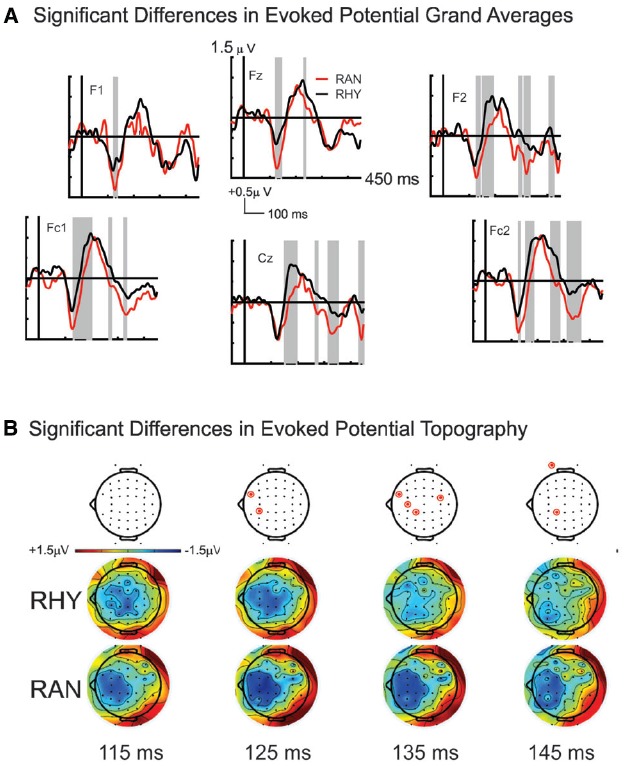
**Grand average ERP responses to sounds in Rhythmic and Random Conditions. (A)** Random (red line) and Rhythmic (black line) grand average responses are shown for fronto-central electrode sites. Significant response differences are shown in shaded gray. **(B)** Scalp topographies over the N1 time window in both Random (bottom plots) and Rhythmic (middle plots) conditions show a fronto-central negativity that is typical of auditory N1 responses. The negativity is stronger in the RAN condition at 125, 135, and 145 ms over several frontal electrodes (denoted in top plots, by red circles).

**FIGURE 6:**
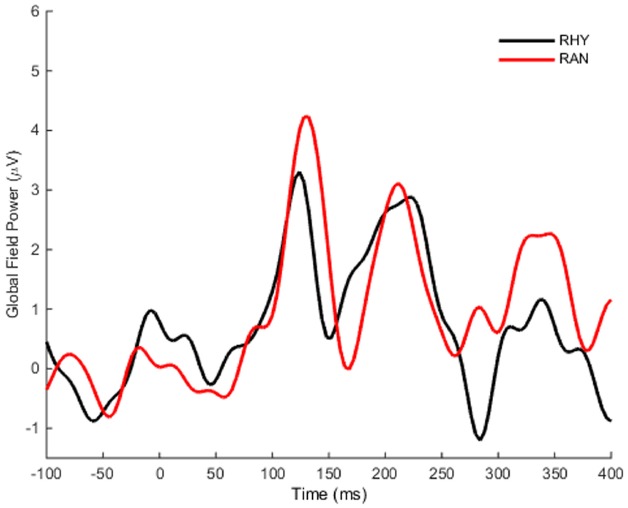
**Grand average global field power.** The mean global field power (GFP) was computed over time for all scalp electrodes. GFP in the Rhythmic condition is shown in black, and the Random response is shown in red.

**FIGURE 7:**
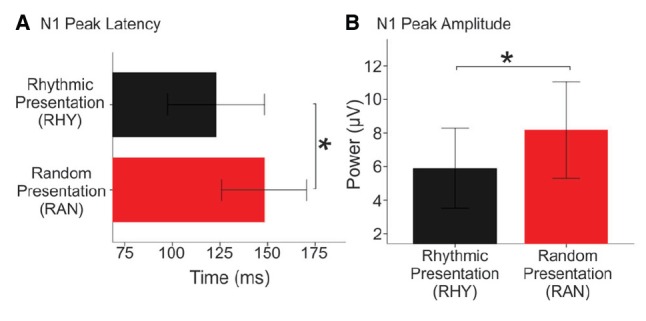
**N1 peak latency and magnitude in Random and Rhythmic Conditions.** Mean N1 latency **(A)** and global field power **(B)** of individual waveforms to tones in Rhythmic (black bars) and Random (red bars) conditions. Error bars show one standard error from the *denotes significance *p* < 0.05.

Our bootstrap analysis of fronto-central waveforms between the two conditions revealed consistent differences (α < 0.001) between random and rhythmic responses in the N1 time window (see gray shaded regions in Figure 5A). Additional regions of differences were also observed at later time points (e.g., over the P2/N2 region), but were less consistent. Bootstrap analysis of scalp topography showed that the negativity in the random condition was significantly larger (α < 0.001) in several fronto-central electrodes during the N1 time window (filled red electrodes, Figure 5B). Analysis of whole scalp GFP (Figure 6) over the N1 time region showed smaller [*t*(9) = 2.74, *p* < 0.04] and faster [*t*(9) = 3.21, *p* < 0.02] N1 peaks in the rhythmic condition compared to random (Figure 7). These measurements of the N1 component that were simultaneously recorded as the fMRI signals thus complement the fMRI data by indicating that the more directly measured electrophysiological activity shows effects in the same direction.

## Discussion

### Behavioral Results

Our behavioral results show more accurate and faster detection of targets embedded in rhythmic (predictable) streams compared to those occurring randomly in time, supporting the improved behavioral efficiency of rhythmic mode processing (Schroeder and Lakatos, 2009). This is in concert with previous findings showing that temporally predictive environmental cues can improve target detection and discrimination (Jones et al., 2006; Praamstra et al., 2006; Ten Oever et al., 2014), see also (Thorne and Debener, 2013). Moreover, we found faster RTs within streams of higher driving rates, which suggests flexibility in our use of temporal cues, such that important information can be detected faster if the environmental rhythm is pushing us to do so.

### BOLD Response

The fMRI results showed a stronger BOLD response bilaterally in superior temporal cortex during the random blocks compared to the rhythmic blocks. These regions are most likely parts of the auditory association cortex, which comprises the superior- and transverse temporal gyrus areas surrounding the primary auditory cortex (Formisano et al., 2003). Although the exact relation between the hemodynamic responses measured with fMRI and the underlying neural activity is still uncertain, a body of evidence shows that hemodynamic responses are strongly positively correlated with broadband high-gamma range neuronal activity, and negatively correlated with lower frequency oscillatory activity (Mukamel et al., 2005; Niessing et al., 2005; Scheeringa et al., 2011).

The stronger fMRI response in auditory areas while participants were attending to the randomly timed tones (i.e., a situation of vigilance) supports our prediction that this temporal context is more metabolically demanding. It is thought that vigilance elicits both the enhancement of continuous high-gamma power as well as low frequency power suppression (Schroeder and Lakatos, 2009). Because high gamma power is strongly correlated with multi-unit neuronal firing, this predicts a stronger BOLD response in vigilant states. Furthermore, it highlights an interesting paradox: the random condition may impose “low-frequency oscillatory suppression” on auditory cortex and this may be accompanied by a larger “evoked N1 response,” perhaps due to an improved signal-to-noise ratio. Congruent with this idea, Niessing et al. (2005) showed that delta-power (1–4 Hz) correlated negatively with hemodynamic response strength. As the driving rate of the sounds in the rhythmic condition in the current study was within the delta range (1.6 and 2.2 Hz), the lower fMRI response during rhythmic vs. random blocks might be expected to accompany enhancement of delta-range oscillatory power.

### Simultaneous EEG

The simultaneously recorded EEG data enable a more direct linking of the fMRI response to neural activity. The increased amplitude and latency of the auditory N1 to the tones in the random blocks supports the interpretation of increased neuronal metabolism due to greater cognitive-energetic effort in the following ways. Peak amplitude and latency are generally thought to reflect the timing and size of neuronal population’s response to incoming stimuli. The N1 amplitude effect demonstrated here is explained by the predictive suppression hypothesis (Lakatos et al., 2009, 2013b) as follows: (1) pure tones activate only a small portion of the tonotopic representations in auditory cortices, and while these regions are entrained to an attended stimulus stream at an optimal excitability phase, all surrounding regions (with different spectral tuning) are entrained to the same rhythmic stream at a low excitability phase (Lakatos et al., 2013a; O’Connell et al., 2014), and (2) the attentional enhancement in the first case is swamped by the attentional suppression of the much larger surround area (Lakatos et al., 2013b). Paradoxically, the hypothesized net effect of stimuli in a rhythmic context is better target detection, but decreased amplitude for attention-related ERP peaks at the scalp (Lakatos et al., 2013b).

In addition, our N1 amplitude finding is compatible with previous reports that the N1 is larger in amplitude when the timing of sound onset cannot be predicted (Lange, 2009). Because the BOLD signal reflects changes in blood oxygenation levels triggered by increased metabolic demands of locally activated neuronal populations (Huettel et al., 2009), predictive suppression should correspond with a decrease in the BOLD activity. This is in fact what we see here: both the BOLD response and N1 amplitude are smaller in the rhythmic condition. While our evidence appears to converge regarding the size of recruited neuronal populations across conditions, the earlier latencies in the rhythmic condition provides additional information regarding response timing. The N1 latency effect suggests that neuronal activity in the rhythmic condition is faster or more strongly time-locked relative to firing in the random condition. Overall, the N1 results show shorter response times and less activation in the rhythmic condition; suggesting more “efficient” neuronal processing compared to responses in the random, unpredictable temporal context.

In addition to exploring processing efficiency across different temporal contexts, we also wanted to examine whether the efficiency flexibly changes by switching contexts, and by repeated exposure to switching contexts. Therefore, we analyzed fMRI activity, RTs to targets and HRs for the eight blocks, which alternated between rhythmic and random sound streams, in each run separately. We found a positive correlations between the fMRI response in the right auditory cortex and RTs for the separate blocks, suggesting that the patterns of hemodynamic and behavioral responses to the alternating temporal contexts was similar. Inspection of the evolution of the fMRI activity in auditory cortex over the course of a run revealed that the higher BOLD response in the random condition did only occur in the beginning of the runs, whereas activity levels in the rhythmic condition stayed constant (Figure 4). A smaller effect, but in the same direction, seems apparent in the RTs (Figure 2B, right panel) This suggests that, interestingly, sound processing and target detection in the random blocks seems more adaptive to repeated exposure, raising the possibility that processing of randomly timed sounds becomes more efficient after repeated exposure to unpredictable sensory information. This may reflect a learning effect, as for rhythmically timed sounds we may already perform at “ceiling” level. More speculatively, it may be possible that the low frequency activity that persists in vigilance conditions (Landau and Fries, 2012; Fiebelkorn et al., 2013), adapts and contributes incrementally to performance. This may be enhanced by the fact that in our current design, rhythmic and random periods alternated, possibly strengthening the reliance on lower frequencies.

### Converging Effects

The combination of fMRI, ERP, and behavioral results support the notion that temporally predictable input streams are processed more efficiently than unpredictable ones, and that the more efficient processing of rhythmic inputs benefits target detection. How the auditory cortex communicates with other components of the distributed activated network during the listening task (Figure 3A) should be investigated in more detail in future studies using connectivity analyses. For example, it will be interesting to explore whether connectivity between the thalamus and auditory cortical areas depends on the rhythmicity of the input (Musacchia et al., 2014).

These results have important implications for natural events with complex temporal structure, such as speech, as they suggest that it is indeed efficient for the brain to tune into a “rhythmic” mode if temporal regularities are present in the environmental input. However, the decrease of the rhythmic-random difference in auditory cortex activation as well as RTs for target detection, suggests that the brain is also able to adapt to situations of irregular temporal input, i.e., becomes more efficient for repeated exposure to such environments, possibly by learning what stimulus features to suppress if certain dimensions remain irrelevant over time. An interesting suggestion is that the adaptation to the unpredictable streams may be facilitated by the intervening rhythmic streams in the current design, which could enhance the predictive suppression mechanism to act also in the less predictable periods. This suggestion needs to be investigated in more detail in follow-up studies.

### Conflict of Interest Statement

The authors declare that the research was conducted in the absence of any commercial or financial relationships that could be construed as a potential conflict of interest.
